# A Tridimensional in vitro Epithelial Model of a Simulated Vaginal Environment for the Study of *Candida albicans* and Herpes Simplex Virus 2 Mono- and Coinfections

**DOI:** 10.1159/int/adgag001

**Published:** 2026-07-31

**Authors:** Stefania Caramaschi, Francesco Ricchi, Arianna Sala, Laura Franceschini, Luca Fabbiani, Marzia Gozzoli, Luca Reggiani Bonetti, Elisabetta Blasi, Claudio Cermelli

**Affiliations:** a Clinical and Experimental Medicine Ph.D. Program, Department of Biomedical, Metabolic and Neural Sciences, Laboratory of Microbiology and Virology, University of Modena and Reggio Emilia, Modena, Italy; b Department of Surgical, Medical, Dental and Morphological Sciences with interest in Transplant, Oncological and Regenerative Medicine, Laboratories of Microbiology and Virology, University of Modena and Reggio Emilia, Modena, Italy; c Department of Medical and Surgical Sciences for Children and Adults, Anatomic Pathology Unit, University of Modena and Reggio Emilia, Modena, Italy; d Molecular Microbiology and Virology Unit, University Hospital Policlinico, Modena, Italy; e Department of Biomedical, Metabolic and Neural Sciences, University of Modena and Reggio Emilia, Modena, Italy

**Keywords:** Herpes simplex virus 2, *Candida albicans*, Polymicrobic infections, Vaginal infections, A431 cells

## Abstract

**Introduction:**

*Candida albicans* (*C. albicans*) and herpes simplex virus 2 (HSV-2) are highly prevalent genital pathogens that often coexist, potentially influencing mutual replication and host responses. Building on our recent work employing mono- and coinfections in A431 epithelial cells, we developed a three-dimensional (3D) in vitro model using inert scaffolds and the presence of a synthetic vaginal fluid (SVF) to better mimic vaginal epithelium.

**Methods:**

A431 cells were cultured on inert 3D scaffolds, with or without SVF for 5 days, and then infected with *C. albicans* and/or HSV-2. Cell viability was assessed by LDH release, fungal burden by CFU assay, and viral load by PCR. Histological and immunohistochemical analyses were performed to evaluate epithelial structure, pathogen presence, and IL-6 production.

**Results:**

The A431 cells formed a multilayered epithelium on scaffolds; yet, mature epithelial phenotype markers were not detected. SVF enhanced fungal growth and viral replication during coinfection. *C. albicans* significantly reduced epithelial viability, whereas HSV-2 showed minimal effects; dual infection caused slightly additive damage. IL-6 was produced in both mono and double infection.

**Conclusion:**

The 3D scaffold-based model supports polymicrobial infection. Nevertheless, the lack of cell epithelial phenotype highlights the need of further strategies to more closely replicate the in vivo vaginal mucosal environment.

## Introduction

Genital tract infections represent a critical public health issue, with notable implications for both individual morbidity and broader epidemiological burden [1–3]. Among the most frequently encountered pathogens are *Candida albicans (C. albicans)* and herpes simplex virus 2 (HSV-2), which exhibit distinct pathogenic mechanisms but often affect overlapping anatomical sites [3–7].

*C. albicans*, a polymorphic fungal organism, is part of the normal human microbiota, colonizing mucosal surfaces such as the gastrointestinal tract, oral cavity, and vaginal epithelium [2, 8]. Under conditions of host dysregulation, particularly immunosuppression, fungal overgrowth may result in mucocutaneous or invasive candidiasis [8–10]. Vulvovaginal candidiasis, the most prevalent manifestation in women, is clinically characterized by pruritus, burning sensation, erythema, and a thick, whitish discharge [6, 11]. These symptoms may partially overlap with the prodromal or acute phases of HSV-2 infection, complicating differential diagnosis, especially when the herpetic vesical lesions are in the vaginal cavity [12, 13]. Lifetime prevalence of vulvovaginal candidiasis is estimated to be as high as 75% in women, with recurrent forms affecting 20–25% [14].

HSV-2 is the etiologic agent of genital herpes, a condition typified by vesiculo-ulcerative lesions of the external genitals, although subclinical or asymptomatic presentations are common, complicating accurate prevalence estimation [13, 15]. Following primary infection, HSV-2 establishes latency within the sacral dorsal root ganglia, with potential reactivation triggered by physiological or pathological stressors such as immunosuppression, psychological stress, and coexisting infections [12]. Epidemiological data indicate that HSV-2 seroprevalence dramatically changes according to age range, being approximately 0% up to the age of 14 years and then progressively increasing mainly starting from the age of 20 years (7.5%–10%). The peak of HSV-2 positivity is reached at 49 years (21%–26%), and afterward, the epidemiologic values remain stable or undergo a light decrease (20%–25%) [16–18]. However, HSV-2 prevalence is influenced by geographic, sex-related, and sociodemographic variables [15, 19].

Coinfection by HSV-2 and *C. albicans* is an underexplored but increasingly recognized phenomenon, particularly in immunocompromised patients, where it may exacerbate disease severity and complicate therapeutic management [5, 6]. Evidence suggests a bidirectional interaction between these pathogens, whereby HSV-2-mediated mucosal disruption and local immunodisturbance enhance fungal adhesion and tissue invasion. On a HeLa cell model, HSV-2 was demonstrated to enhance *C. albicans* adhesion and biofilm initiation by decreasing heparan sulfate proteoglycans on cell membranes as well by altering the membrane fluidity induced by IFN produced in response to HSV-2 infection [4, 20]. Inflammatory cytokines released in response to HSV-2 may potentiate *Candida* pathogenicity, contributing to a favorable environment for fungal proliferation [21, 22]. This interplay underscores a synergistic pathogenic relationship, wherein one infectious agent facilitates the persistence or exacerbation of the other [4, 5, 23–25].

Despite limited dedicated studies, the clinical implications of *C. albicans*/HSV-2 coinfection are significant, particularly in cases of recurrent genital herpes [26, 27]. These patients may experience more intense symptomatology, increased frequency of reactivation, and enhanced therapeutic failure. Concurrent antiviral and antifungal interventions may be required, highlighting the need for integrated diagnostic and treatment approaches [3, 5]. Furthermore, the psychosocial burden of chronic HSV-2 infection, often associated with stress-induced immunosuppression, may also predispose to secondary fungal colonization [27]. Elucidating the pathological mechanisms governing this dual infection is essential for advancing diagnostic accuracy, optimizing patient-specific treatment strategies, and ultimately improving prognostic outcomes.

We recently published the results of a study investigating *C. albicans* and HSV-2 mono- and coinfections in the A431 epithelial cell line [28]. It is known that the A431 cells, originating from a human epidermoid carcinoma of the vulvar region, possess the capacity to form either a confluent monolayer (24-hour culture) or a stratified, differentiated multilayer, after 5 days of culture [28, 29]. The latter, named as reconstituted vaginal epithelium (RVE), closely recapitulates in vitro the architecture and barrier characteristics of human vaginal epithelium, even more when a synthetic vaginal fluid (SVF) is added to the culture medium [28]. Expanding upon this model, the present study proposes a three-dimensional (3D) organotypic system, by culturing A431 cells on an inert scaffold matrix, in the presence of SVF. This approach aims to replicate, as accurately as possible, the morphological and functional properties of the native vaginal mucosa, even the spatial interactions between epithelial cells and *Candida*, or HSV-2-infected epithelial cells and *Candida*, thereby enabling in-depth analysis of epithelial pathogen-pathogen interactions, under both mono- and coinfection conditions.

## Methods

### Epithelial Cells

All experimental procedures were carried out using the A431 cell line, derived from a human epidermoid carcinoma in the vulvar region (ATCC CLR-1555). Cells were cultured as previously described [28]. Briefly, cells were maintained in Dulbecco’s Modified Eagle Medium (Sial S.r.l., Rome, Italy) enriched with 2 mm L-glutamine (Gibco, Thermo Fisher Scientific Italia), 100 U/mL penicillin-streptomycin (Lonza Walkersville Inc., MD, USA), 2.5 mg/mL ciprofloxacin (Gibco, Fisher Scientific Italia, Segrate, Italy), and 10% heat-inactivated fetal bovine serum (Sial S.r.l., Rome, Italy), hereafter named as complete medium, while 5% for experimental procedures. The cells were maintained by twice weekly passages and incubation at 37°C in a 5% CO_2_ atmosphere.

### Fungal Strain and Growth Conditions

The *C. albicans* SC5314 reference strain (ATCC MYA-2876) was used for all experiments. The fungal strain was preserved at −80°C in Microbank^®^ cryovials (ProLab Diagnostics, Richmond Hill, ON, Canada) as previously described [30]. Prior to use, the frozen stock was thawed and cultured in yeast extract peptone dextrose broth (Scharlab S.L., Barcelona, Spain), followed by incubation at 37°C under aerobic conditions and agitations (120 rpm), for 24 h and next subcultured in Sabouraud dextrose agar (Oxoid, Thermo Scientific Italia) through weekly passages. Only fungal cells in the exponential phase of growth were employed in the experiments.

### HSV-2 Strain

A clinical isolate of HSV-2, initially identified using a monoclonal antibody and subsequently adapted to laboratory conditions through over 50 serial passages in Vero cells, was employed. Viral stocks were prepared by centrifuging lysates from HSV-2-infected A431 cells which had been cultured for 2–3 days, following previously established protocols [31]. Prior to experimental use, the virus was titrated on A431 cells, aliquoted, and stored at −80°C. All experiments were conducted using the same viral batch to ensure consistency.

### Establishment of a 3D in vitro Model

A431 cells were cultured to establish a 3D in vitro model on inert scaffolds. The Alvetex^®^ scaffold plate wells (REPROCELL, Glasgow, Scotland) were at first hydrated with ethanol 70% (vol/vol) (Sigma-Aldrich), washed with warm PBS, and next conditioned with complete medium. Subsequently, the A431 cells were seeded onto the scaffolds at a density of 4 × 10^5^ cells/scaffold. The plates were incubated for 1 h at 37°C in a humidified atmosphere containing 5% CO_2_. Finally, 1 mL of complete medium was added at each well and the plates were incubated for 5 days, with a complete medium change performed on day 3.

### Scaffold Handling and Processing within the Anatomical Pathology Laboratory

To perform histological analysis, the scaffolds were removed from the wells and placed in 10% buffered formalin-based fixative, as routinely done in diagnostic pathology to preserve cellular and structural integrity. After 24-h fixation, samples underwent standard histopathological processing, at the pathology laboratory according to accredited procedures for dehydration, clearing, and embedding. Paraffin-embedded blocks were then sectioned into thin tissue slices (4 μm) using instrumentation compliant with clinical practice standards. Sections were mounted on glass slides for routine histological evaluation and on positively charged slides for immunohistochemistry. Hematoxylin-eosin (H&E) staining, performed under internal quality-controlled protocols, was used for general morphological assessment. Additional histochemical and immunohistochemical (IHC) stains were applied to paraffin sections, corresponding to the H&E slide that exhibited the highest cellularity. Staining was performed by the BenchMark IHC/ISH automated system (Ventana Medical Systems) following standard antigen retrieval protocols. Antibody binding was visualized using the Ventana OptiView Universal DAB IHC Detection Kit with 3,3′-diaminobenzidine as the chromogen. Slides were then counterstained with hematoxylin II, Mayer-modified (Roche Diagnostics – Ventana Medical Systems), to highlight cellular morphology.

### IHC Staining for Cytokeratin 5/6 and Epithelial Membrane Antigen

Detection of cytokeratin 5/6 was carried out using a mouse monoclonal primary antibody (clone D5/16B4, Roche Diagnostics – Ventana Medical Systems, Tucson, AZ, USA). Detection of epithelial membrane antigen (EMA) was performed using a mouse monoclonal primary antibody (clone E29, Roche Diagnostics – Ventana Medical Systems, Tucson, AZ, USA). If less than 10% of cells showed brown membranous and/or cytoplasmic staining, the expression of the corresponding antibody was classified as negative.

### Infection Protocol Applied to the 3D in vitro Model

Scaffold-based cultures were exposed or not to SVF (25% vol/vol), following a previously established protocol [28] and then infected with *C*. *albicans* by adding 250 μL of fungal suspension per well, corresponding to a multiplicity of infection (MOI) of 0.5:1 (fungal cells:epithelial cells). After a 3-h incubation at 37°C, 250 μL of HSV-2 suspension was added to achieve a final MOI of 0.1:1 (virus:cells), bringing the total working volume to 1 mL/well. The cultures were then incubated for additional 21 h at 37°C in a humidified atmosphere containing 5% CO_2_, before proceeding to downstream analyses.

### Histochemical Staining for Fungal Organisms

Detection of fungal structures was performed using the Grocott staining method, a silver impregnation technique routinely employed for the visualization of fungal cell walls. The procedure was carried out according to standard histochemical protocols for silver-based staining.

### IHC Staining for HSV-2

HSV-2 detection was performed using a mouse monoclonal primary antibody (clone 3C5.2, Cell Marque, Rocklin, CA, USA). IHC staining and counterstaining were carried out using the same procedures previously described. Cells exhibiting brown nuclear or nuclear-cytoplasmic staining were considered positive for the virus. Each sample was evaluated and classified as negative (0% stained cells) or positive, according to the presence of stained cells.

### Pathogen Quantification

The quantification of *C*. *albicans* within the scaffolds was performed using a semiquantitative method, assessing the presence of the microorganism, the amounts of hyphae, and the hyphal length by microscopic observation. Each sample was assigned a score of 0 if *Candida* was absent, or 1, 2, or 3 based on increasing amounts or lengths of hyphae. All results were collected in a table, from which a graph was generated.

HSV-2 viral load quantification was performed by a commercial real-time PCR system (HSV2 ELITe MGB^®^ Kit, Elitech, Turin, Italy), following DNA extraction with a commercial kit (Elite InGEnius SP200, Elitech, Turin, Italy), in accordance with the manufacturer’s instructions. The PCR protocol included a decontamination step at 50°C for 2 min, an initial denaturation at 94°C for 2 min, followed by 45 cycles of 94°C for 10 s, 60°C for 30 s, and 72°C for 20 s.

### IHC Staining for Interleukin-6 Detection

Detection of interleukin-6 (IL-6) was carried out using a mouse monoclonal primary antibody (clone BSB-140, Bio SB, Santa Barbara, CA, USA). IHC staining and counterstaining were performed using the same procedures previously described. Cells exhibiting brown membranous and/or cytoplasmic staining were considered positive for the corresponding antibody. Each sample was evaluated and categorized as negative (<10% positive cells), partially positive (10–80%), or displaying diffuse expression (>80%). Staining intensity was semiquantitatively scored as weak (1), moderate (2), or strong (3).

### Statistical Analysis

Statistical analysis was performed on the results obtained from viral load quantification. The Shapiro-Wilk test was used to analyze data distribution within each experimental group. Subsequently, for the DNA copies/mL viral load statistical analysis was performed by one-way ANOVA test; Tukey’s multiple comparisons were chosen as post hoc tests. To analyze the data in terms of the presence of SVF or *C. albicans*’ roles in virus replication, an unpaired t test was used. All statistical analyses were carried out using GraphPad Prism 10 software. Values of *p* < 0.05 were considered statistically significant.

## Results

### Histological Evaluation of the A431 Epithelial Cells Cultured within Inert Scaffolds

After 5 days of incubation, the A431 cells diffusely and homogeneously adhered to the matrix, in both the presence and the absence of SVF, as shown in Figure 1. The cells adhered firmly to the underlying matrix, forming a continuous layer without evident gaps or clustering. Cellular morphology appeared preserved, with clearly defined cell borders and nuclei, indicating a healthy and stable epithelial arrangement. This homogeneous colonization of the matrix suggests that the culture conditions effectively supported cell attachment and initial stratification, thus validating the suitability of the model for further experimental applications.

**Fig. 1.**
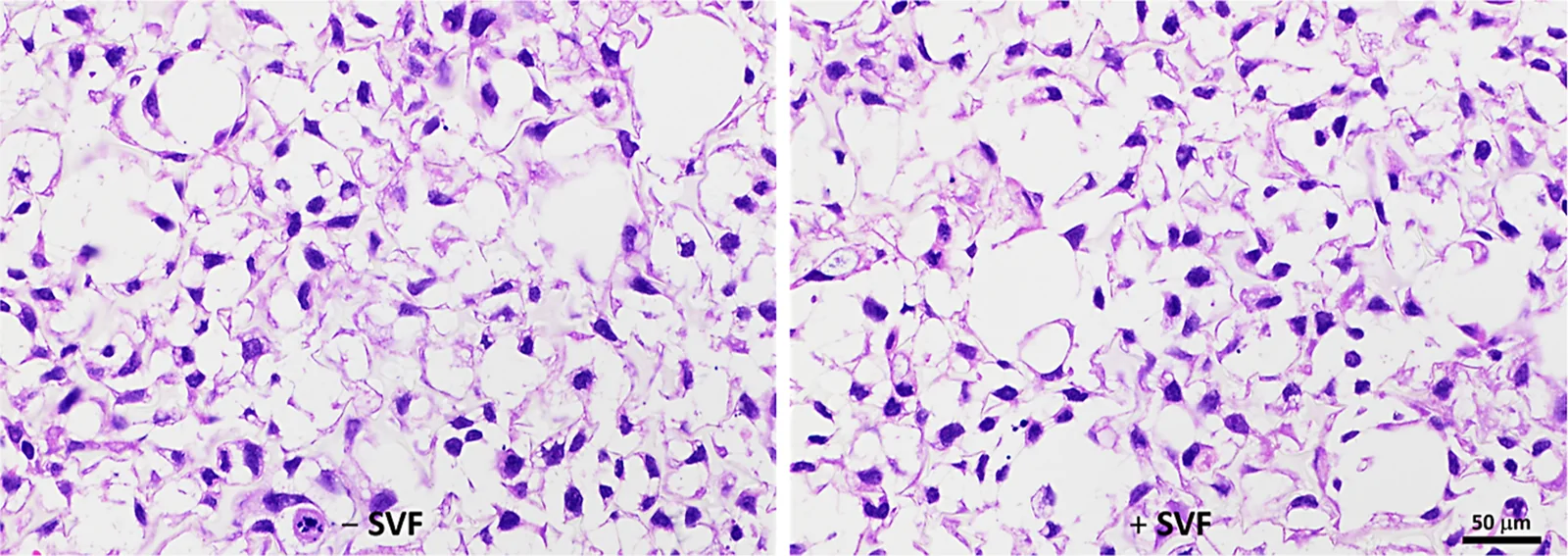
Morphological aspect of the A431 cells cultured into inert scaffolds (H&E. staining, 40× magnification). Histological evaluation revealed that A431 cells were diffusely and uniformly distributed across the surface of the scaffold, both in the presence and in the absence of SVF.

### IHC Evaluation of the Epithelial Phenotype of A431 Cells under Different Culture Conditions

IHC analysis for CK 5/6 was performed, revealing negative staining in the 3D-cultured A431 cells, irrespective of the presence of SVF, indicating the lack of detectable cytokeratin expression, as illustrated in Figure 2. This result was in contrast with what had been observed in RVE grown in chamber slides, according to a recently established protocol [28].

**Fig. 2.**
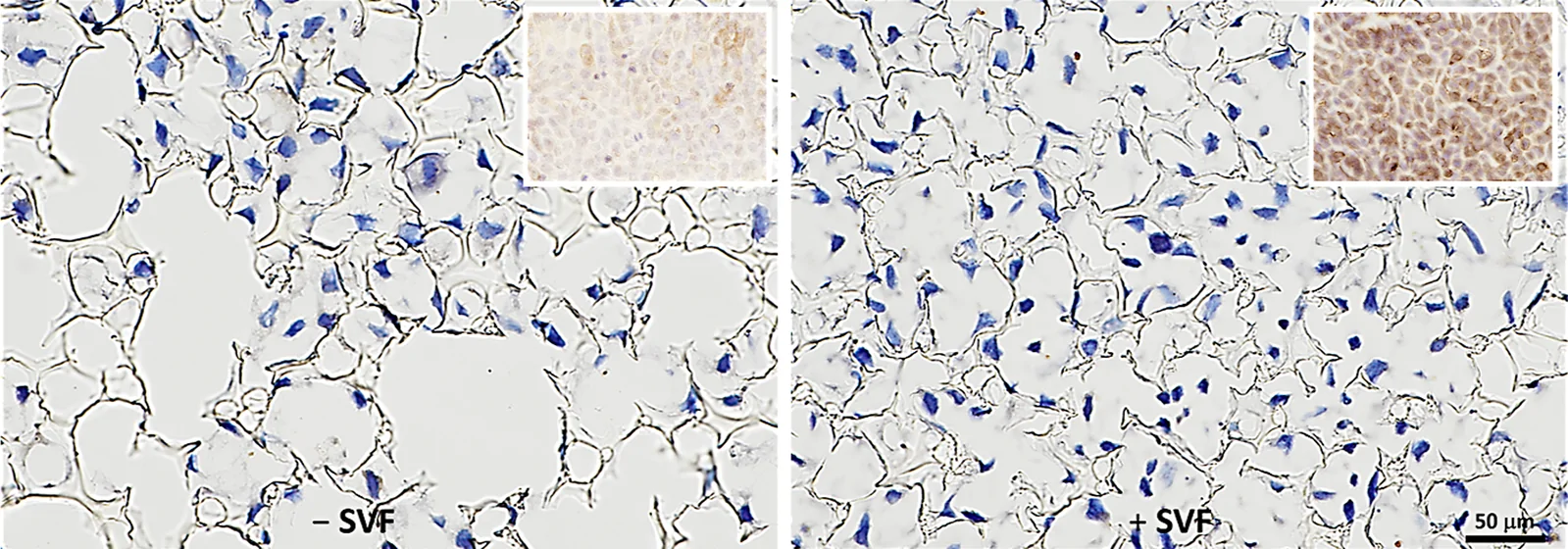
CK 5/6 expression in A431 cells, with or without SVF (IHC staining, 40× magnification). IHC revealed that the A431 cells cultured on scaffolds were CK 5/6 negative, in contrast to what had been observed in RVE grown in chamber slides, which showed diffuse expression of such marker, with a stronger intensity observed with SVF (inserts in the corners).

To further verify the epithelial phenotype, IHC staining for EMA was performed. Consistent with the findings observed for CK 5/6, also the EMA expression remained undetectable in the scaffold-cultured cells, regardless of SVF exposure. No membranous or cytoplasmic staining was detected in any of the evaluated samples (Fig. 3).

**Fig. 3.**
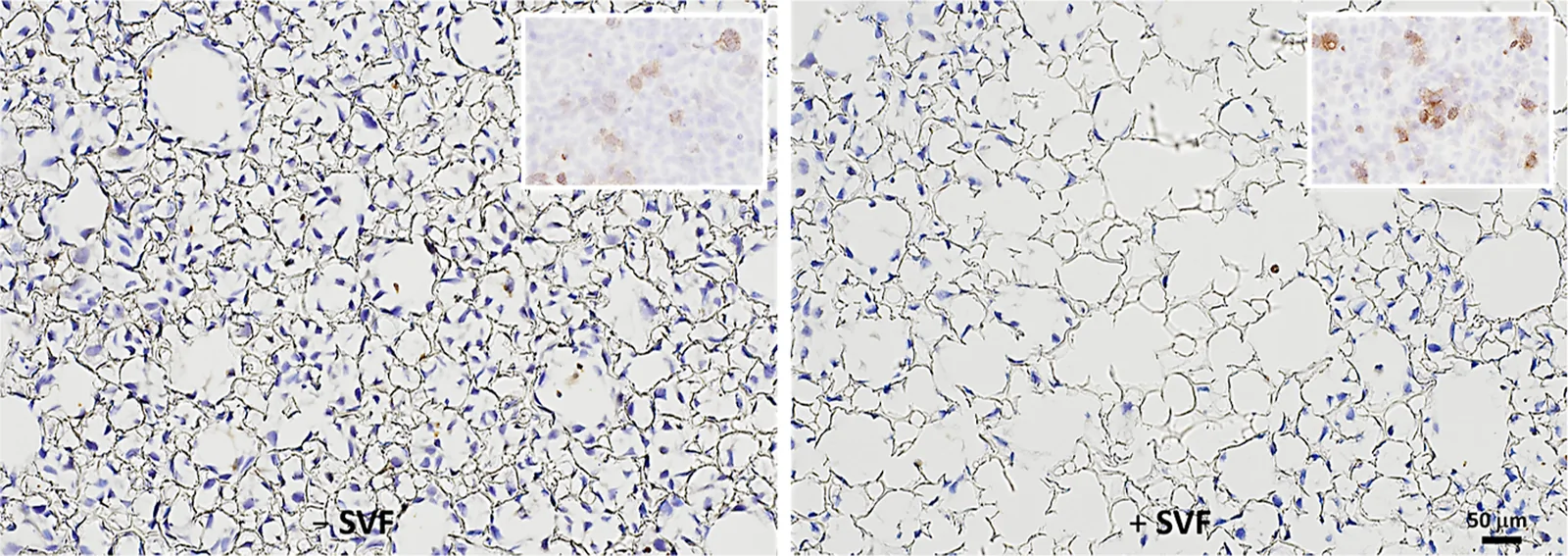
EMA expression in A431 cells, with or without SVF (IHC staining, 20× magnification). IHC showed that the A431 cells grown on scaffolds were EMA negative, while positive expression of the marker had been observed in RVE cultured in chamber slide, with a slightly higher intensity observed with SVF (inserts in the corners).

### Growth of *C. albicans* in A431 Scaffold with or without SFV

The A431 cells, cultured into the 3D scaffolds for 5 days, were infected with *C. albicans* at an MOI of 0.5:1 (fungal cells:epithelial cells) in either the presence or the absence of SVF and then microscopically observed. As depicted in Figure 4, the amount of *Candida*, observed as both yeast and hyphal forms, was markedly increased in the presence of SVF. In contrast, only a few hyphae were detected in scaffolds cultured without SVF.

**Fig. 4.**
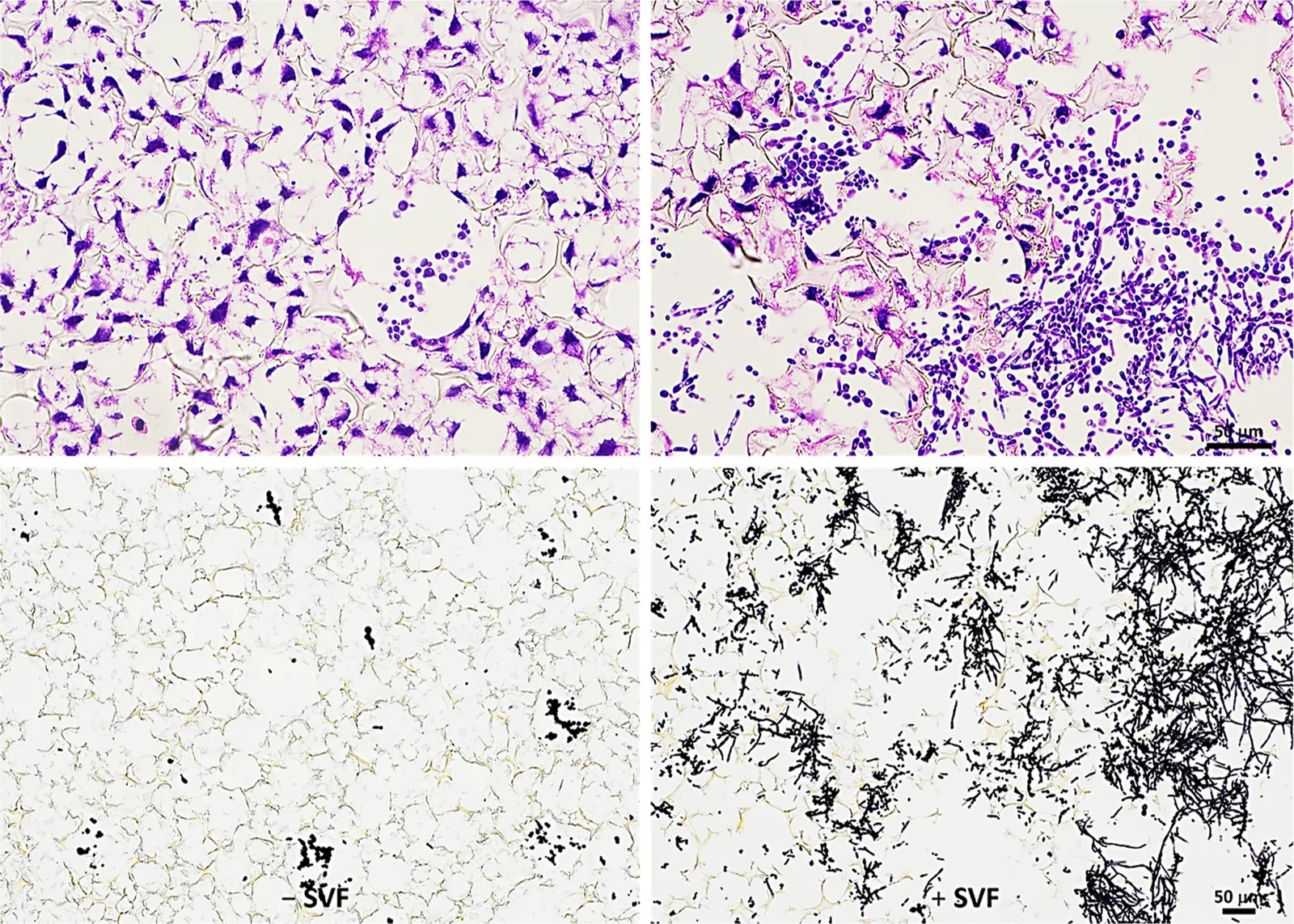
Morphological aspect of the A431 cells, cultured into inert scaffolds and infected with *C. albicans*, in the presence or absence of SVF (upper panels: H&E. staining, 40× magnification; lower panels: GROCOTT staining, 20× magnification). The epithelial cells grew adherently to the trabeculae of the scaffold, forming a 3D structure, while *C. albicans* grew mainly in the intertrabecular spaces. Both H&E and IHC show that the amount of *C. albicans* was increased in the presence of SVF.

Semiquantitative analysis confirmed that SVF markedly enhanced *C. albicans* growth and filamentation within the 3D scaffolds. As depicted in Figure 5, the fungal burden was higher in the presence of SVF, both in samples infected with *C. albicans* alone and in those coinfected with HSV-2. Similarly, the quantity of hyphae and their average length were substantially increased when SVF was added to the culture system, indicating a strong SVF-dependent promotion of fungal filamentation. In contrast, conditions without SVF, whether with *C. albicans* alone or combined with HSV-2, displayed noticeably lower fungal load, reduced hyphal numbers, and shorter hyphae (Fig. 5).

**Fig. 5.**
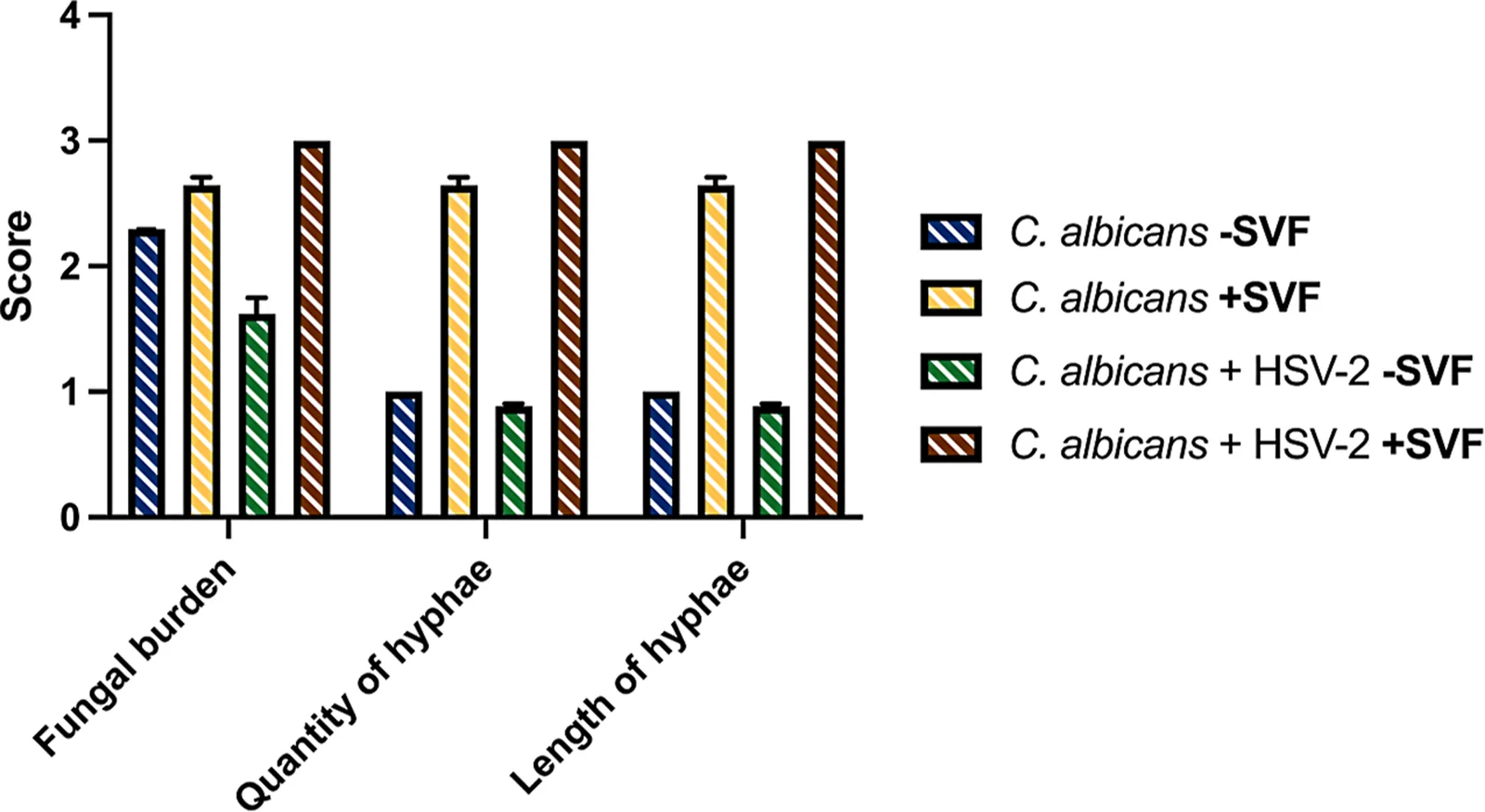
Semiquantitative evaluation of *C. albicans* load/filamentation onto 3D cultured A431 cells. The graph illustrates the total fungal burden, the quantity of hyphae, and the hyphal length under the different experimental conditions. In the presence of SVF, *C. albicans* growth, hyphal formation, and length were enhanced, while HSV-2 had no effects on any parameter.

### HSV-2 Infection in the Presence or Absence of SFV

As depicted in Figure 6, H&E staining revealed distinct morphological alterations in A431 cells following HSV-2 infection, with notable differences depending on the presence of SVF. In the presence of SVF, the infected cells displayed moderate cytopathic changes. Conversely, when SVF was not added to the culture system, these cytopathic effects appeared markedly increased. The cells exhibited cytoplasmic vacuolization, nuclear enlargement, and condensed chromatin, consistent with heightened viral-induced damage (upper panels). Overall, these observations indicate that SVF reduces the cytopathic damage of A431 cells to HSV-2 infection, resulting in less severe morphological disruption within the 3D scaffold environment. IHC staining using a monoclonal antibody was performed to detect HSV-2 in A431 cells cultured in 3D scaffolds, with or without SVF. The analysis revealed a reduction in HSV-2-infected cell positivity in the presence of SVF (lower panels).

**Fig. 6.**
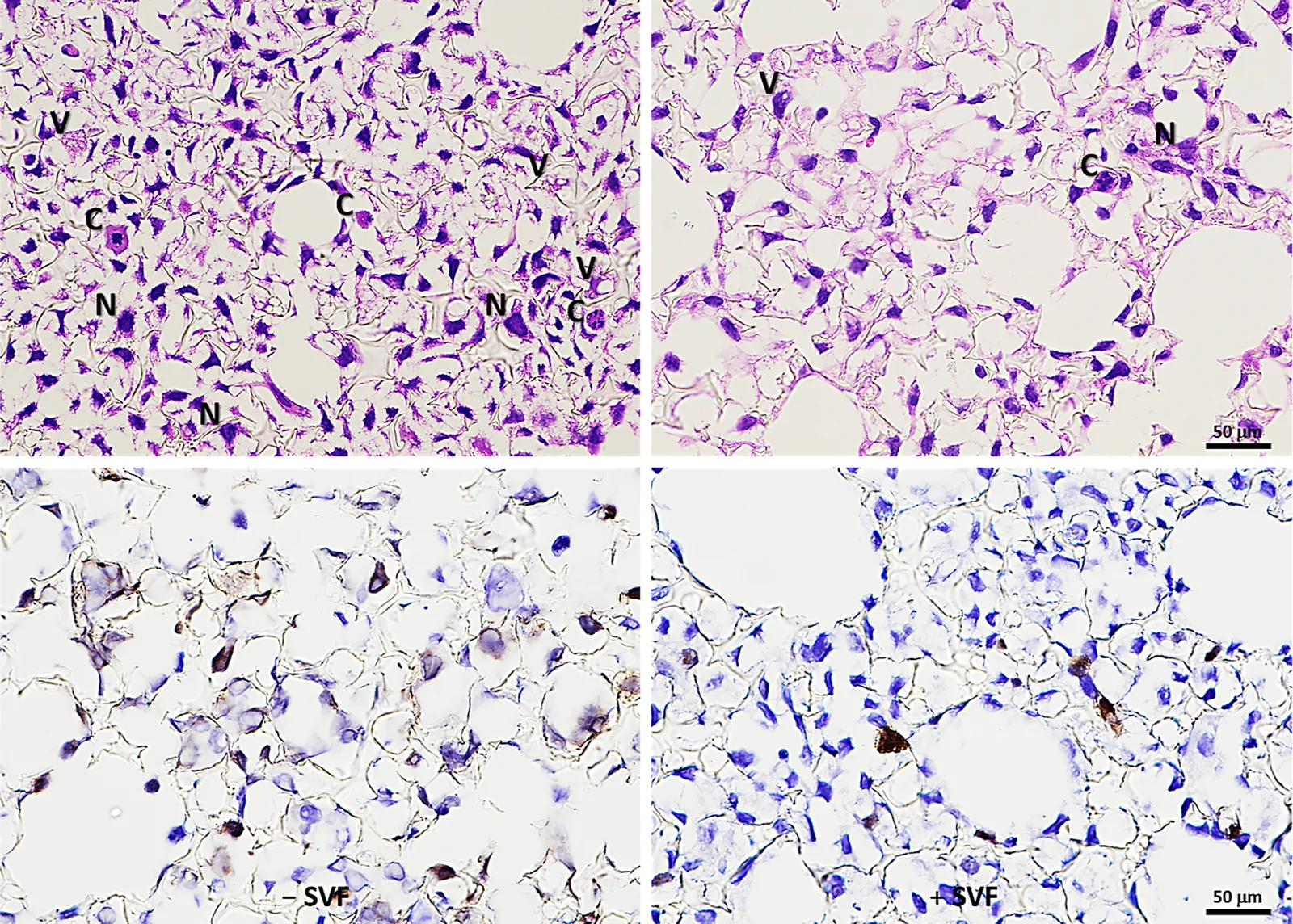
Morphological alteration in A431 cells infected with HSV-2, with or without SVF (upper panels: H&E. staining, 40× magnification; lower panels: IHC staining, 40× magnification). H&E. staining reveals HSV-2-associated cytopathic effects, including cytoplasmic vacuolization (labeled **V**), nuclear enlargement (**N**), and chromatin condensation (**C**), predominantly in the absence of SVF. IHC images show A431 epithelial cells positive for anti-HSV-2 antibody, mainly without SVF.

Figure 7 illustrates the morphological changes (upper panels) and the IHC staining for HSV-2 (lower panels) in A431 double infected cells. In particular, the IHC analysis reveals a higher number of HSV-2-positive cells in the presence of SVF.

**Fig. 7.**
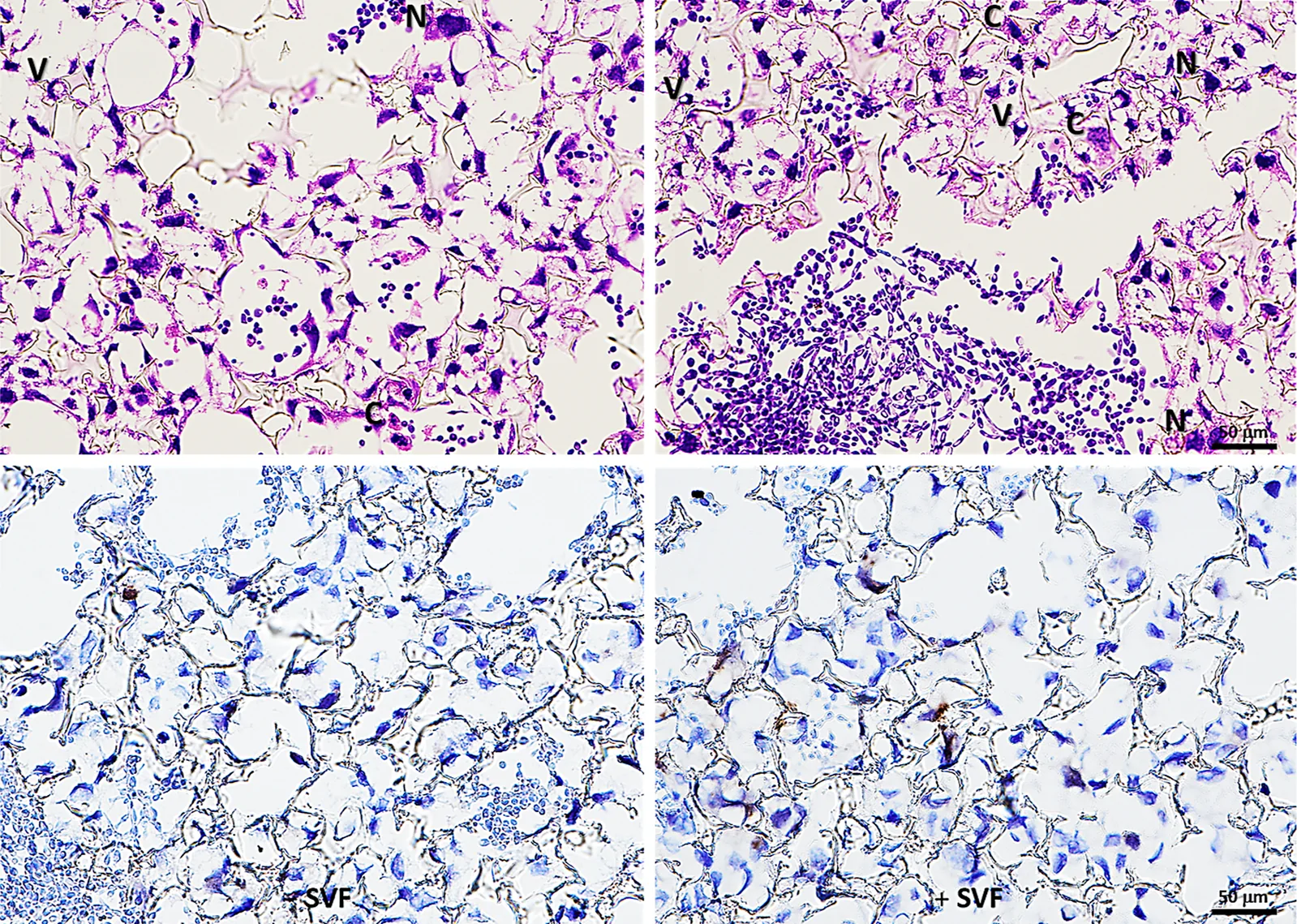
Morphological aspect of the A431 cells, cultured into inert scaffolds, and exposed to the dual infection, *C. albicans* and HSV-2, in the presence or absence of SVF (upper panels: H&E. staining, 40× magnification; lower panels: IHC staining, 40× magnification). H&E. staining shows HSV-2-associated cytopathic effects, including cytoplasmic vacuolization (labeled **V**), nuclear enlargement (**N**), and chromatin condensation (**C**), predominantly in the presence of SVF. IHC images illustrate A431 epithelial cells positive for anti-HSV-2 antibody, in slightly higher numbers with SVF.

Figure 8a describes the levels of HSV-2 DNA copies, determined using RT-PCR. The amount of viral DNA ranged between 2.5 × 10^6^ copies/mL (in the coinfection condition without SVF) and 5.1 × 10^6^ copies/mL (in the coinfection condition with SVF), with no significant changes with respect to the presence of SVF. Yet, in the absence of SVF, the viral infectious titers decreased in cells that had also been infected with *C. albicans* (4.8 × 10^6^ vs. 2.5 × 10^6^ copies/mL) while, in contrast, in the presence of SVF, the HSV-2 DNA copies approximately doubled in dually infected cells with respect to A431 cells infected with the virus alone (2.9 × 10^6^ vs. 5.1 × 10^6^ copies/mL). Figure 8b shows the Log_2_ fold change of viral DNA production in relation to the presence or absence of SVF in order to identify its role in virus replication. A reduction in the number of HSV-2 DNA copies was observed in the presence of SVF in single infection, while in double infection there was an increase; statistical analysis of these data indicated a significant role (*p* < 0.01) of SVF. Figure 8c illustrates the same analysis with respect to *C. albicans*; statistical analysis showed a significant role of *C. albicans* in HSV-2 replication (*p* < 0.01).

**Fig. 8.**
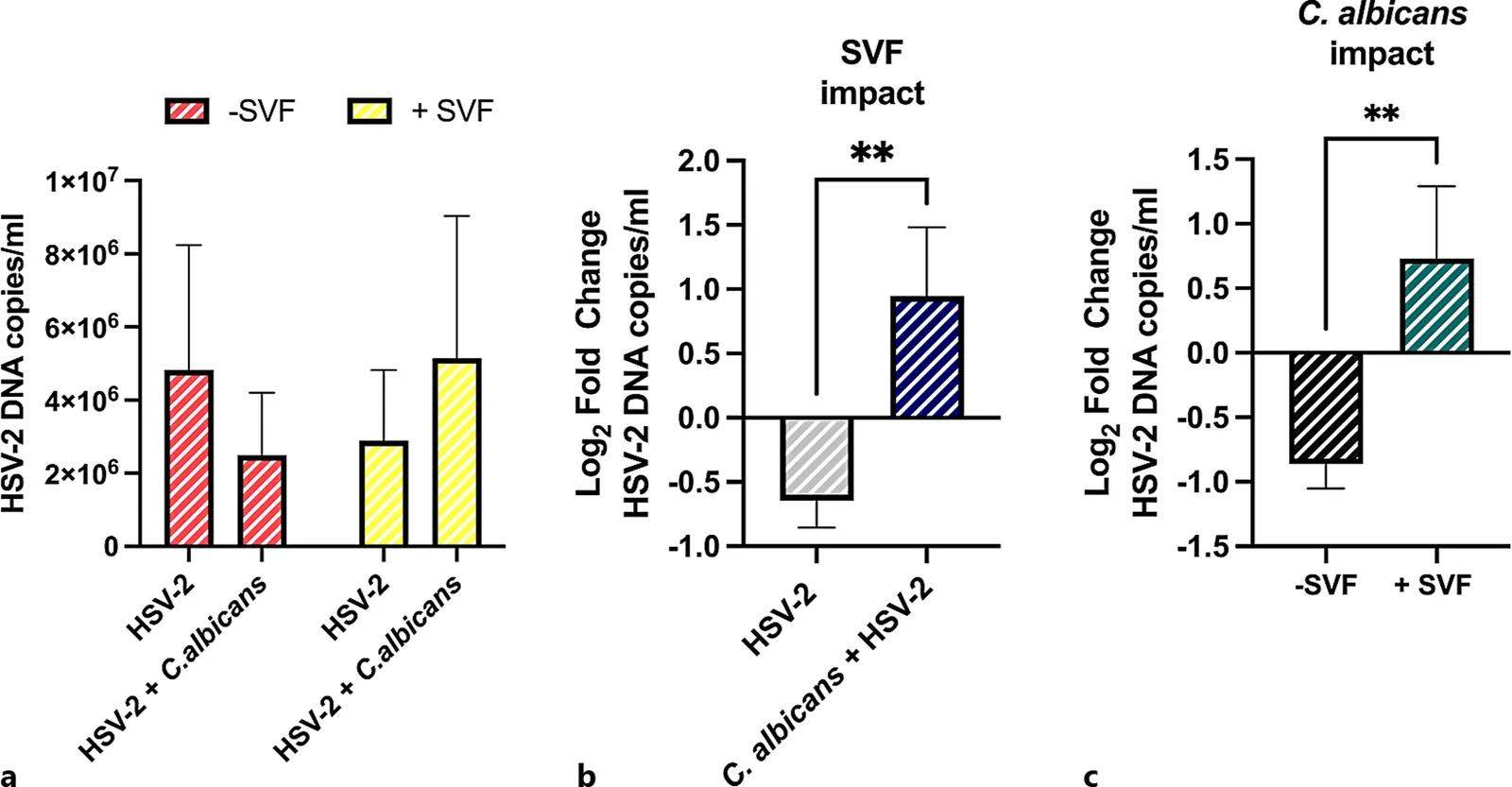
Analysis of viral load in 3D cultured A431 cells. The graphs illustrate HSV-2 DNA copy levels under different experimental conditions, determined by RT-PCR (**a**); the impact of SVF on viral DNA, which decreased in single infection and increased in double infection (**b**); the impact of *C. albicans* on viral DNA, which decreased without SVF and increased with SVF (**c**). The values of **p < 0.01 were considered statistically significant.

### IHC Evaluation of IL-6 in A431 Cells under Different Culture Conditions

As depicted in Figure 9, IHC staining revealed differential IL-6 expression among the different experimental conditions, which tended to be higher in A431 cells infected with HSV-2. In the setting of single viral infection, IL-6 immunoreactivity was evident in both the presence and the absence of SVF. In contrast, during double infection, a higher number of IL-6-positive cells were observed in samples with SVF compared with those without SVF. In cells infected with *C. albicans* alone, IL-6 staining was generally weak, whereas it increased in the presence of SVF. Overall, these findings indicate an increased IL-6 expression in the presence of SVF.

**Fig. 9.**
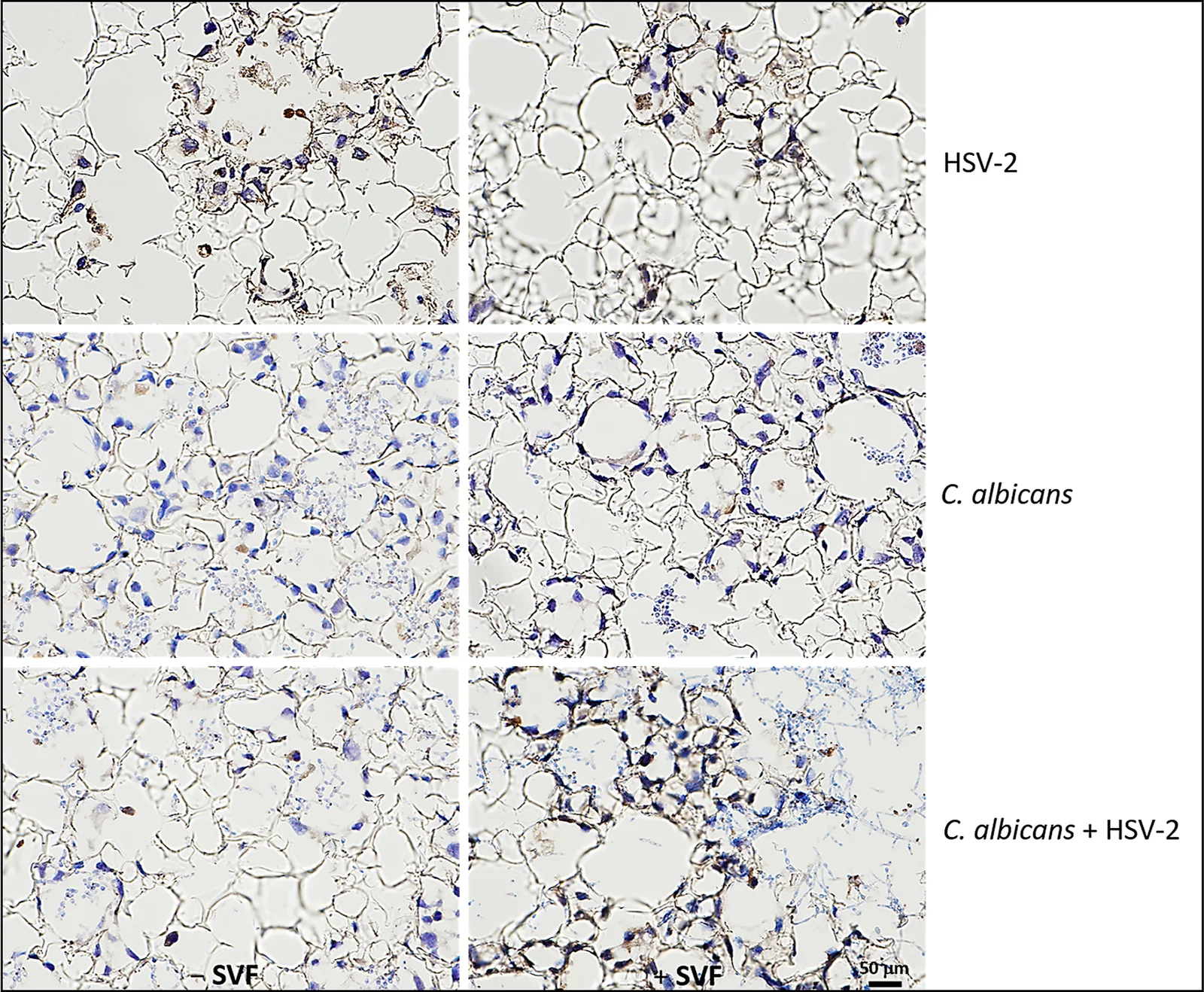
IL-6 expression in A431 cells cultured into inert scaffolds with or without SVF (IHC staining, 40× magnification). IHC revealed differential IL-6 expression among the different experimental conditions of infection. An enhanced number of positive cells were observed in samples infected with HSV-2, particularly in cases of dual infection and in the presence of SVF.

## Discussion

In this study, we set a scaffold-based 3D human cell culture system as a novel in vitro model that closely simulating the vaginal mucosa may allow investigations on the interplay among A431 epithelial cells, *C. albicans*, and HSV-2 during polymicrobial infection [4, 5, 20, 23–25]. *C. albicans* and HSV-2 are frequently involved in vaginal diseases; although the pathogenic mechanisms of each of the two are largely known, their either synergistic or antagonistic interactions in double infections are still little studied. In this respect, an in vitro model faithfully simulates the vaginal mucosal environment, bypassing the numerous problems intrinsic of the animal and HSV-2 during epithelial cell infection, employing the vaginal epithelioma-derived A431 cell line. In this model, the cells were grown as RVE, namely, a 5-day multilayer of cells that had reached a high level of differentiation and structuration, thus closely simulating the vaginal environment [28]. A crucial step forward in mimicking the in vivo situation was achieved by adding SVF to the cultures that indeed provided a relevant modulation of the events occurring during the mono- and double infection, with *C. albicans* paving the way to HSV-2. In the present work, in an attempt to further improve this model, the A431 cells were cultured for 5 days onto 3D synthetic scaffolds and subsequently were infected with one or both pathogens, in the presence or in the absence of SVF. In line with our previous results [28], the present findings strengthen the relevance of SVF, given the increased fungal load and filamentation as well as the viral-mediated epithelial cell damage with respect to the presence of the SVF. Unexpectedly, the 3D model here above described also highlights an apparent incongruence between the efficient cellular adhesion to the scaffold and the little to undetectable acquisition/expression of epithelial differentiation markers. As documented by the H&E staining, the A431 cells adhere diffusely and uniformly to the scaffold surface after 5 days of culture, forming a continuous and morphologically preserved epithelial layer. This finding indicates that the scaffold provides a valid and supportive physical environment that allows tight cell attachment and persistence/viability. However, despite the successful adhesion, the adherent cells fail to proceed toward differentiation, as documented by the fact that the CK 5/6 and EMA markers are undetectable. The expression of both markers remains consistently negative regardless of SVF exposure. Importantly, this finding is in contrast with what is observed using the 2D chamber slide model, where the CK 5/6 is induced, mostly after SVF addition [28]. These somehow unexpected results obtained employing the 3D epithelial model agree with previous studies showing that these scaffolds effectively preserve stemness by mimicking the natural stem cell niche architecture [32, 33]. In the absence of specific exogenous differentiation stimuli, the scaffold’s precise geometry exerts controlled mechanical constraints on the cell nucleus, which inhibit spontaneous differentiation. This mechanical conditioning maintains a high expression of pluripotency markers and prevents the premature lineage commitment typically observed in traditional 2D cultures [32, 33].

Taken together, these findings suggest that while scaffold architecture favors cell adhesion and early 3D organization, it may lack key biochemical or mechanical cues necessary to trigger and sustain epithelial differentiation. This conclusion should be carefully considered as a limitation when interpreting the in vitro data produced employing scaffolds. Accordingly, any statement on infection-related outcomes, considering the impact of either mono- or dual infections onto 3D epithelial cell models, should be taken with special caution, since it is well known that epithelial maturity strictly influences host-pathogen interactions and scaffolds happen to prevent it.

A major finding of this study is the strong enhancement of *C. albicans* proliferation and filamentation in the presence of SVF. Both histological and semiquantitative analyses reveal a markedly enhanced fungal burden in SVF-supplemented scaffolds, accompanied by an increase in the number and length of hyphae. Interestingly, this effect persists under HSV-2 coinfection, underscoring a robust SVF-dependent stimulation of fungal growth. Several features of SVF, such as a high glucose concentration and the acid pH, that are known to characterize the in vivo vaginal environment likely contribute to the enhanced fungal fitness detected in our experiments. As previously observed also using the scaffold-free RVE model of A431 cells [28], this increase in fungal activity linked to SVF did not show any differences related to the presence or absence of HSV-2. This finding seems to be in contrast with literature data showing that HSV-2 infection increases the adhesiveness of *C. albicans*, albeit with a different cell model and experimental protocols [4, 20].

Thus, in line with our previous paper employing the 2D model of RVE [28], the present data further strengthen the relevance of including SVF in in vitro studies, as a critical element to mimic as close as possible the in vivo environmental condition peculiar of the vaginal mucosa. The impact of SVF is also evident in the context of HSV-2 infection. Morphological alterations of the epithelial cells caused by HSV-2 are consistently more evident in the absence of SVF, whereas the presence of SVF mitigates cytopathic effects, resulting in less pronounced cytoplasmic vacuolization, nuclear enlargement, and condensed chromatin. IHC analysis further supports this trend, showing a reduction in HSV-2-positive cells when SVF is present. The molecular quantification of HSV-2 DNA confirms these observations; in the case of single infection, DNA production is reduced in the presence of SVF, while, when *C. albicans* is present, DNA levels also increase. These observations suggest that SVF may exert a partial protective or modulatory effect on epithelial cells, during HSV-2 infection. The SVF inhibitory activity may be potentially mediated by alteration of virus-cell interaction, likely through interference with cell surface molecules involved in virus attachment and entry. As a matter of fact, the SVF contains different molecules that can exert this effect. First, urea is demonstrated to inhibit HSV-2 replication [34]. Other SVF components that could likely explain this phenomenon are glucose at high concentration and glycerol which can modify the structure and quantity of heparan sulfate proteoglycans: these molecules have a key role in HSV-2 attachment and entry in the target cells [35]. Moreover, glycerol influences cell membrane fluidity which could in turn alter endosome formation for virus entry [36]. Finally, the SVF acid pH can modify receptor binding kinetics. Although the precise mechanisms remain to be clarified, these findings highlight the complex interplay between vaginal microenvironment and viral pathogenesis.

The dual infection with *C. albicans* and HSV-2 appears to produce additive effects on cell alterations, although not markedly beyond the impact of the individual pathogens. Although the cells appear only mildly morphologically altered in the double infection, *C. albicans* has a significant impact on HSV-2 replication in the presence of SVF, the viral DNA being doubled. A possible explanation may be the consumption of SVF by fungal overgrowth, thus resulting in reduced protective effects against HSV-2 infection.

Finally, we investigated IL-6 expression in the infected cells, by IHC. The role of this cytokine as a molecular bridge between innate and adaptive immunity is critical in the host defense against both viral pathogens and fungal agents like *C*. *albicans*. As a pleiotropic cytokine, IL-6 is essential for orchestrating B-cell maturation and Th17 cell differentiation, the latter being the primary immunological axis for mucosal fungal clearance [8, 37, 38]. HSV-2 is a strong IL-6 inducer at mucosal level, even more efficient than HSV-1 triggering a significantly more robust cytokine surge [19, 39]. In our in vitro model, we observe an increased expression of IL-6 in the presence of SVF with both pathogens; yet, HSV-2 induces a higher cytokine production than *C. albicans*, with the highest positivity rate being observed in the double infection. We envisage that this HSV-2-driven IL-6 production may have profound implications with respect to the dual infection and co-pathogenesis. Since IL-6 is indispensable for the Th17 response, its overproduction during an HSV-2 infection may paradoxically disrupt the fine balance required for effective *Candida* clearance. While a baseline level of IL-6 is protective, the exacerbated response induced by HSV-2 may lead to immunopathology, compromising mucosal barrier integrity and enhancing host susceptibility to fungal colonization [40]. So, if on one hand, *C. albicans* acts as a harbinger for HSV-2 infection, creating a permissive milieu that facilitates the subsequent entry and replication of HSV-2, on the other hand, we can speculate a host favorable immunomodulation by HSV-2 in *C. albicans* persistence and replication. Nevertheless, these results should be interpreted with caution considering the lack of epithelial phenotype of the scaffolded cells and therefore a direct correlation between the results obtained using this in vitro model and the in vivo situation could be controversial.

In summary, this work establishes a 3D scaffold-based culture system capable of supporting epithelial cell adhesion and microbial infection, while revealing important limitations in epithelial differentiation. The strong enhancement of *C. albicans* growth and the modulatory effects on HSV-2 infection induced by SVF emphasize the relevance of incorporating physiologically meaningful environmental factors in infection models. In this context, *C. albicans* and HSV-2 exhibit a bidirectional synergism, where each pathogen actively promotes the environmental conditions necessary for the other’s persistence/replication. However, a significant caveat of this study is the lack of a mature epithelial phenotype in the 3D scaffold-cultured cells. Consequently, this system may not fully recapitulate the physiological microenvironment of the human vaginal mucosa, suggesting that the observed interactions among A431 cells, *C. albicans*, and HSV-2 should be interpreted with caution when extrapolated to in vivo conditions. In this perspective, stem cell cultures induced to vaginal epithelium differentiation could likely be a more suitable substrate, but their use for microbiological investigations is still limited. Future studies are necessary to explore novel strategies for promoting epithelial cell maturation within the scaffolds; likely, specific matrix modifications or prolonged culture times may allow improvement of the physiological fidelity of 3D mucosal models and, in turn, may facilitate advanced studies on vaginal mono- and polymicrobial infections under conditions that at best mimic in vivo exposure.

## Statement of Ethics

The A431 cell line and the strain of *C. albicans* used in this work were purchased from ATCC, while the HSV-2 strain is part of the virus collection of our laboratory. Ethical approval for the use of these is not required in accordance with local/national guidelines.

## Conflict of Interest Statement

The authors have no conflicts of interest to declare.

## Funding Sources

This research was funded by the Italian Ministry of the University – MUR (Grant PRIN2022 – UA.20 – CUP E53D23001270006 – project title “Innovative mucosal models as prototypes for in-depth studies on the pathogenesis of monomicrobial and polymicrobial infections.” Prot. 2022_LS6 – 2022C7JE2F). The funder had no role in the design, data collection, data analysis, and reporting of this study.

## Author Contributions

Conceptualization: Elisabetta Blasi and Claudio Cermelli; methodology: Stefania Caramaschi, Francesco Ricchi, Luca Fabbiani, Laura Franceschini, Marzia Gozzoli, and Arianna Sala; software: Francesco Ricchi; validation: Claudio Cermelli, Stefania Caramaschi, and Francesco Ricchi; formal analysis, investigation, and data curation: Stefania Caramaschi and Francesco Ricchi; writing – original draft preparation: Stefania Caramaschi and Claudio Cermelli; writing – review and editing: Stefania Caramaschi, Francesco Ricchi, Luca Reggiani Bonetti, Claudio Cermelli, and Elisabetta Blasi; supervision: Claudio Cermelli; project administration and funding acquisition: Elisabetta Blasi. All authors have read and agreed to the published version of the manuscript.

## Data Availability

All data generated or analyzed during this study are included in this article. Further inquiries can be directed to the corresponding author.
